# Macrophage-driven exosomes regulate the progression of cardiovascular disease

**DOI:** 10.3389/fphar.2025.1563800

**Published:** 2025-04-30

**Authors:** Liao Qi, De-Zhu Luo, HuLi Li, JianWen Yan, WenJie He

**Affiliations:** ^1^ Pengzhou Hospital of Traditional Chinese Medicine, Pengzhou, China; ^2^ Department of Anesthesiology, West China Hospital of Sichuan University, Chengdu, Sichuan, China; ^3^ West China Hospital of Sichuan University, Chengdu, Sichuan, China

**Keywords:** exosomes, extracellular vesicles, cardiovascular disease, macrophage polarization, inflamation

## Abstract

Exosomes, as vital mediators of intercellular communication, play a critical role in the progression of cardiovascular disease (CVD). Recently, macrophage-derived exosomes (Mφ-Exos) have garnered increasing attention because of their significant potential in early diagnosis, pathological processes, and therapeutic applications for CVD. Exosomes contain diverse nucleic acids (e.g., miRNAs, mRNAs, and long noncoding RNAs (lncRNAs)) and proteins, which serve as specific biomarkers that regulate various stages of CVD. For example, miRNAs encapsulated within exosomes (e.g., miR-21, miR-133a, and miR-155) are closely associated with atherosclerosis, myocardial infarction, coronary artery disease, and stroke, and changes in their abundance can serve as diagnostic and prognostic indicators. Additionally, the composition of Mφ-Exos, including miRNAs, lipids, and proteins, plays a significant role in the initiation, progression, and inflammation of CVD. Research on Mφ-Exos provides new directions for early diagnosis, mechanistic exploration, and novel therapeutic targets in CVD. However, challenges remain regarding exosome isolation and identification technologies. Future studies need to further explore the biological properties of exosomes and develop more efficient, economical, and straightforward isolation methods. This review summarizes the multifaceted regulatory roles of Mφ-Exos in CVD, encompassing key processes such as inflammation, angiogenesis, metabolism, and cell death. Research has shown that M1-Exos promote the progression and exacerbation of CVD through pro-inflammatory and pro-fibrotic mechanisms, while M2-Exos demonstrate significant therapeutic potential via anti-inflammatory, pro-angiogenic, and metabolic reprogramming pathways. These findings not only reveal the complex mechanisms of Mφ-Exos in CVD but also provide new perspectives and potential targets for early diagnosis and precision treatment of the disease.

## 1 Introduction

In China, the mortality rate of CVD remains high, with increasing incidence and mortality attributed to unhealthy lifestyles, a high prevalence of CVD risk factors, and an aging population (The, 2023).

The immune response plays a pivotal role in the onset and progression of CVD, where macrophages, as key immune cells, profoundly influence the cardiovascular system by regulating inflammation and tissue repair (Roy et al., 2022). During cardiovascular injury, newly recruited macrophages—mobilized via C-C chemokine receptor 2 (CCR2)—become the primary effector cells following the death of resident macrophages (Li L. et al., 2023; Heidt et al., 2014). These macrophages not only engulf tissue debris and release proinflammatory cytokines but also regulate extracellular matrix (ECM) production, promote cell proliferation, and drive angiogenesis, playing a central role in inflammatory responses (Li L. et al., 2023). Furthermore, macrophages release various mediators that engage in complex crosstalk with other cell types, influencing endothelial cell generation, fibrosis, and immune cell activity and thereby significantly impacting the progression of CVD (Peng et al., 2023; Chen R. et al., 2024).

Extracellular vesicles (EV), which have emerged as novel mediators of intercellular communication, have received considerable attention in recent years. Exosomes, a subtype of EV with a diameter of 30–150 nm, carry proteins, lipids, and nucleic acids and play important roles in cell signalling, immune regulation, and tissue repair (Pluchino and Smith, 2019; Mousavi et al., 2022; Lu et al., 2019). Studies have shown that the components of exosomes reflect the physiological states of their parent cells, making them potential biomarkers for early diagnosis, prognosis, and therapeutic monitoring of CVD (Neves et al., 2023). Furthermore, exosomes can traverse biological barriers, serving as effective drug delivery vehicles capable of transporting therapeutic molecules to target cells (Lu et al., 2019; Shao et al., 2018).

Macrophage-derived exosomes (Mφ-Exos) are emerging as a research hotspot in the context of CVD. These exosomes play pivotal roles in maintaining cardiovascular homeostasis and driving pathological progression by modulating myocardial repair, vascular remodelling, and tissue regeneration. Additionally, exosomes secreted by different macrophage subtypes exhibit distinct functional differences in CVD. For example, M2a macrophages promote angiogenesis and tissue repair during diabetic wound healing (Jiang et al., 2024), whereas the M2b subtype performs immunomodulatory functions under interleukin-4 (IL-4) stimulation (Yang et al., 2019). The M2c subtype is notable for its role in regulating inflammation and restoring ECM homeostasis (Liu et al., 2023). Foam cell-derived exosomes influence the progression of atherosclerosis (AS) by modulating the proliferation of human umbilical vein endothelial cells (HUVECs) (Li et al., 2024; Ley et al., 2011). These studies highlight the potential of Mφ-Exos to regulate complex intercellular communication networks in CVD, providing a theoretical foundation for exploring novel therapeutic strategies.

Therefore, in-depth investigations into the biological properties and functional mechanisms of Mφ-Exos in CVD not only contribute to elucidating CVD pathophysiology but also pave the way for the development of exosome-based therapeutic tools and biomarkers (Zhang Z. et al., 2023; Quaglia et al., 2020). This research direction holds significant promise for advancing precision medicine technologies, ultimately improving the diagnosis and treatment of patients with CVD (Burnap and Mayr, 2021; Du et al., 2023).

## 2 General information on exosomes

### 2.1 History of exosomes

The term *“exosome”* was first used to describe unknown vesicles released from cultured cells that carry 5′-nucleotidase activity (Trams et al., 1981). As research has progressed, the definition of exosomes has gradually expanded. In the 1980s, researchers first used *exosomes* to refer to small membrane vesicles released during the differentiation of reticulocytes, which typically range in diameter from 30 to 100 nm (Johnstone et al., 1987). By the mid-1990s, exosomes were reported to be secreted by B lymphocytes and dendritic cells, and they were proposed to play a significant role in immune regulation, even being considered potential carriers for antitumour immune responses (Raposo et al., 1996; Zitvogel et al., 1998). The phenomenon of exosome secretion has subsequently been reported in nearly all cell types, and its key role in intercellular communication under both physiological and pathological conditions has been widely confirmed (Colombo et al., 2014).

Moreover, the concept of microvesicles was introduced to describe subcellular structures originating from platelets, initially referred to as “platelet dust” (Wolf, 1967). Early studies focused primarily on the role of microvesicles in the coagulation process, but it was later discovered that microvesicles also play a role in intercellular communication. In cancer cells, these vesicles are often referred to as *oncosomes* (Al-Nedawi et al., 2008). Compared with exosomes, microvesicles have a broader size range, ranging from 50 nm to 1,000 nm, and, in some cases, exceed 10 μm. Their formation involves outwards budding and fission of the plasma membrane (PM), releasing them into the extracellular space (Tricarico et al., 2017).

Recent studies have shown that different cell types can regulate the biogenesis of EV (including exosomes and microvesicles) in response to their physiological state, releasing vesicles with specific lipid, protein, and nucleic acid compositions (Colombo et al., 2014). This regulatory mechanism not only reflects the physiological state of the parent cell but also plays an important role in intercellular signal transduction and interaction, providing new insights into the complexity of cell communication.

### 2.2 Formation and Function of Exosomes

As critical delivery vehicles for intercellular communication, exosomes participate in the regulation of tissue, organ, and organismal functions through autocrine and paracrine pathways. Cells regulate short-range intercellular signalling via the endoplasmic reticulum (ER)-Golgi secretory pathway (the ER is responsible for the synthesis and initial modification of proteins and lipids, and it participates in the formation of multivesicular body (MVB) precursors through interactions with the endosomal system. The Golgi apparatus further modifies and sorts these molecules, directing them to MVB via ESCRT-dependent or -independent pathways, ultimately promoting the maturation and release of exosomes) and secretory autophagy (Hosseini-Beheshti et al., 2012), promoting the release of proteins such as cytokines and growth factors (Kuo et al., 2022). To achieve specific and targeted signal delivery, cells encapsulate selected proteins, lipids, RNA, and DNA into membrane-bound vesicles, with diameters ranging from 30 to 150 nm, known as exosomes.

Exosomes are a subtype of small EV that differ from microvesicles—another type of vesicle that forms via outwards budding of the PM (50–1,000 nm in diameter). In contrast, exosomes originate from the inwards budding of the membrane of the MVB (van Niel et al., 2018; Mathieu et al., 2021). When MVB fuse with the PM, they release their intraluminal vesicles (ILVs) as exosomes into the extracellular space. The cell type and endogenous state of MVB influence the specificity of the cargo and surface proteins on exosomes (Sung et al., 2020).

The diversity of exosome cargo provides the basis for the specificity of intercellular communication. The composition and quantity of secreted exosomes are regulated by physiological (Todkar et al., 2021; Ferreira et al., 2022), pathological (Zhang et al., 2021; Lee et al., 2023), and therapeutic conditions (Dar et al., 2021).

#### 2.2.1 Biogenesis pathways of exosomes

**FIGURE 1:**
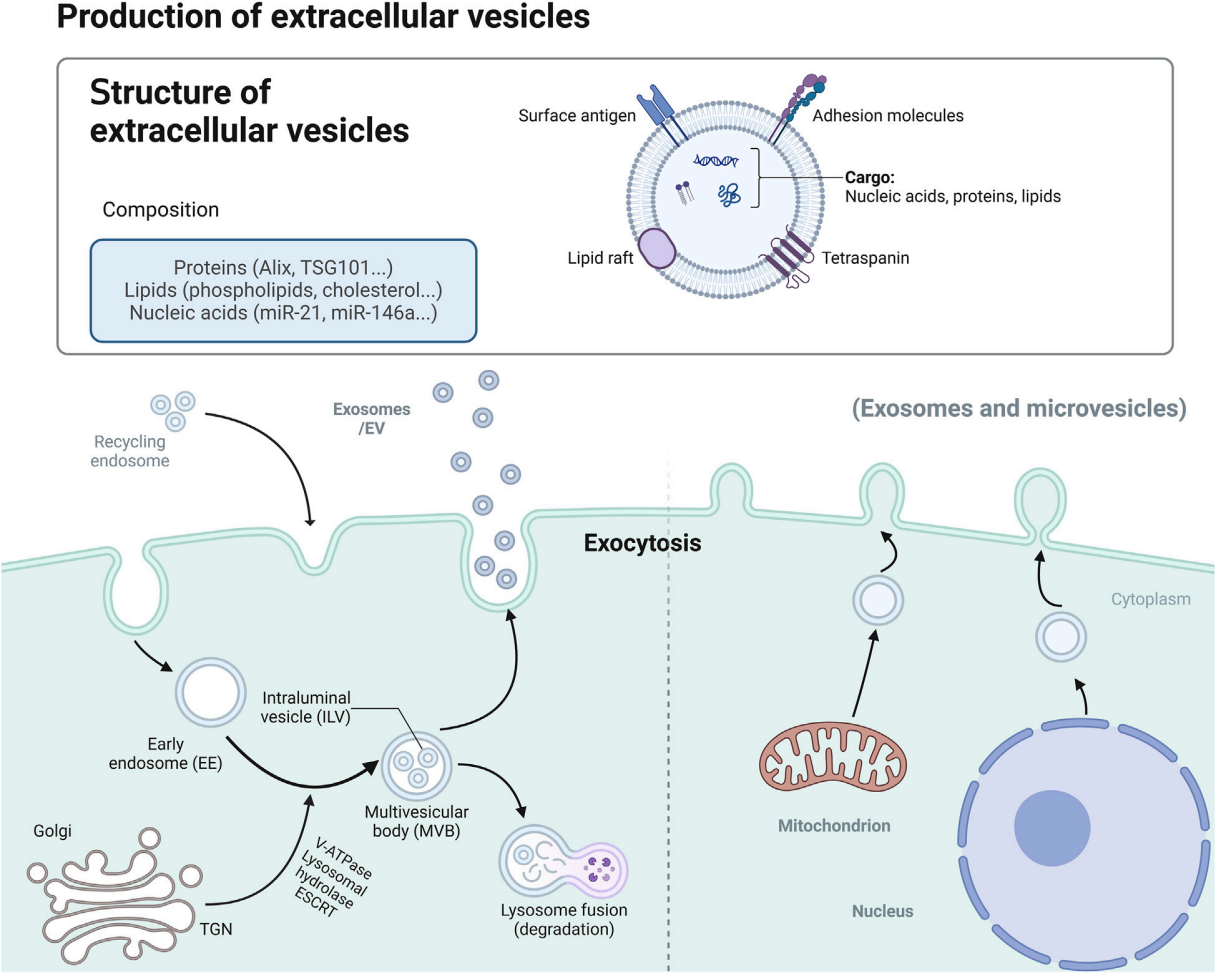
Formation and Function of Exosomes. Extracellular vesicles form when the plasma membrane invaginates, creating endosomes that develop into MVB. The TGN provides components for MVB maturation. MVB either degrade in lysosomes or release vesicles through membrane fusion. Vesicles can also form from mitochondrial or nuclear membranes. Key proteins like ALIX and TSG101 help in vesicle formation and cargo sorting. ALIX ALG-2-interacting protein X, MVB Multivesicular bodie, TSG101 tumor susceptibility gene 101 protein, TGN Trans-Golgi Network.

The formation of exosomes begins with the endocytosis of the PM and involves two steps of membrane budding. In the first step, the PM invaginates under the regulation of asymmetric distributions of multiproteins (such as clathrin) and lipids (such as ceramide and cholesterol), forming *endosomes* (Hubert et al., 2020; Arya et al., 2024; Farsad and De Camilli, 2003). This process is typically governed by specific membrane proteins and cytoskeletal reorganization (Ferguson et al., 2012; Vietri et al., 2020). In the second step, the limiting membrane of the endosome buds inwards, generating intraluminal vesicles and forming MVB (van Niel et al., 2018; Babst, 2011). The formation of ILVs can occur through endosomal sorting complexes required for transport (ESCRT)-dependent or ESCRT-independent pathways, which must overcome the energetic barriers of membrane deformation. This process relies on lipid segregation and local aggregation of protein molecules (Vietri et al., 2020; Babst, 2011; Wenzel et al., 2018; Liese et al., 2020) (Figure 1).

The maturation of MVB is closely linked to the *trans-Golgi network* (TGN), which facilitates cargo sorting by supplying V-ATPase, lysosomal hydrolases, and ESCRT components. Selective cargo modifications, such as phosphorylation, ubiquitination, and glycosylation, further influence ILV biogenesis (Woodman and Futter, 2008; Kwon et al., 2016; Saadi et al., 2018; Giovannone et al., 2017; Huang and Wang, 2017). Finally, mature MVB are sorted for degradation in lysosomes or for exosome release at the PM. Exosome release depends on membrane fusion proteins, such as *SNARE* (Villarroya-Beltri et al., 2016; Han et al., 2017). Notably, the intracellular cholesterol level and its distribution across different organelles also influence the fate of MVB through *membrane contact sites* (MCSs) (Albacete-Albacete et al., 2020; Barman et al., 2022; Verweij et al., 2022) (Figure 1).

#### 2.2.2 Typical pathways of exosomes formation

In addition to the traditional PM- and endosome-derived pathways, exosomes can also form through membrane budding from other organelles (Vietri et al., 2020; Kalluri and LeBleu, 2020). These findings suggest that the mechanisms of exosome biogenesis may vary among cell types and are closely associated with specific physiological and pathological conditions (Lee et al., 2023).

Mitochondria: Mitochondria can generate exosomes enriched with mitochondria-specific proteins and RNAs through membrane budding. These mitochondrial exosomes play critical roles in intercellular signalling. Under conditions of oxidative stress or cellular damage, the release of mitochondria-derived exosomes increases, and these exosomes may contain molecules associated with apoptosis and inflammatory responses (Suh and Lee, 2024; Ke et al., 2020) (Figure 1).

Nuclear Membrane: Under specific conditions, the nuclear membrane can generate vesicles by budding its inner and outer membranes. These vesicles are rich in molecules associated with gene expression and cell cycle regulation and can interact with MVB to generate exosomes (Ritter et al., 2022). Nuclear membrane-derived exosomes may play important roles in the tumor microenvironment and cellular phenotype transitions (Yokoi et al., 2019; Paskeh et al., 2022) (Figure 1).

Recycling Endosomes: As the primary pathway for recycling endocytosed cargo back to the PM, recycling endosomes also participate in exosome formation. They regulate exosome release by transporting internalized materials back to the PM through rapid or slow recycling pathways (Xu et al., 2024).

Additionally, *secretory autophagy* and autophagolysosomal pathways also act as supplementary mechanisms in exosome biogenesis, particularly in processes associated with autophagy, apoptosis, and inflammatory responses (Arya et al., 2024).

### 2.3 Composition and isolation techniques of exosomes

#### 2.3.1 Composition of exosomes

The composition of exosomes is highly heterogeneous, reflecting the cell type and functional state of their origin. While exosomes derived from the same cell type typically contain similar proteins, nucleic acids, and lipids, recent studies have demonstrated that their components are influenced not only by cell type but also by cellular origin, state, and environmental factors (Wen et al., 2019). These components exhibit distinct functions under various physiological and pathological conditions, particularly during different stages of CVD (Wang Y. et al., 2020).

Exosomes carry diverse nucleic acid molecules, including microRNA (miRNA), messenger RNA (mRNA), long noncoding RNA (lncRNA), and circular RNA (circRNA). These molecules play critical roles in regulating gene expression and have promising potential as biomarkers for research and clinical applications (Chen et al., 2019; Oliveira et al., 2020). Exosomes are also rich in membrane transport-associated proteins, such as RAB GTPases, annexins, flotillins, and proteins associated with MVB biogenesis, including ALG-2 interacting protein X (Alix) and tumor susceptibility gene 101 protein (TSG101) (Lv et al., 2020; Zhang et al., 2022; Lee et al., 2024). In addition, transmembrane proteins such as CD9, CD63, and CD81, as well as heat shock proteins (HSP60 and HSP90), play key roles in exosome biological functions (Yu et al., 2022).

Lipids constitute another significant component of exosomes and include mainly sphingomyelin (SM), phosphatidylserine (PS), phosphatidylinositol (PI), phosphatidic acid (PA), ceramide, and cholesterol (Ghadami and Dellinger, 2023). The lipid bilayer of exosomes not only protects their internal cargo from degradation but also facilitates intercellular signalling (Li et al., 2019).

The composition of Mφ-Exos has unique biological properties. The key proteins include MVB-associated proteins (e.g., Alix and TSG101) and membrane proteins, which are critical for exosome biogenesis and intercellular communication (Sung et al., 2020; Gurung et al., 2021). Lipid components such as phospholipids, cholesterol, and wax esters also regulate exosome stability and interactions with target cell membranes (Lee et al., 2024; Skotland et al., 2017). Among the exosome miRNAs, miR-21 and miR-146a are abundantly expressed in Mφ-Exos and have been shown to play significant roles in CVD (Zhang S. et al., 2023). For example, miR-21 promotes cardiomyocyte survival by inhibiting apoptotic signalling pathways, thereby influencing post MI repair processes (Kura et al., 2020; Surina et al., 2021). Moreover, miR-146a plays a key role in modulating inflammatory responses and maintaining immune homeostasis (Mortazavi-Jahromi et al., 2020) (Figure 1).

#### 2.3.2 Methods for exosomes isolation

A variety of methods are available for exosome isolation, including differential ultracentrifugation (UC), immunoaffinity capture, microfluidics, polymer precipitation (PEG precipitation), ultrafiltration (UF), and size exclusion chromatography (SEC) (Wu et al., 2023; de Freitas et al., 2021). Each technique has distinct advantages and limitations in terms of efficiency, purity, and operational complexity, making it crucial to understand their differences when research findings are integrated.

Differential ultracentrifugation (UC) is a widely used method that employs high centrifugal forces (100,000–150,000 × g) to isolate exosomes from cellular debris, larger EV, and other particles (Yang et al., 2020). Although UC is time-consuming and labor-intensive, it remains an effective choice for isolating large volumes of exosomes, especially in clinical sample processing. However, achieving absolute exosome purity is challenging, as contaminants such as exosome aggregates, protein complexes, or viruses may coisolate, compromising the accuracy of the results (de Freitas et al., 2021).

In recent years, immunoaffinity capture and microfluidic technologies have emerged as powerful exosome isolation strategies because of their high specificity and sensitivity. These methods can selectively capture target exosomes, but limitations such as marker dependence and high costs remain concerns (Greening et al., 2015). Polymer precipitation (PEG precipitation) is a simple and efficient technique that separates exosomes via nontoxic, water-soluble polymers (Jalaludin et al., 2023). While this method is easy to perform and does not require expensive equipment, its low specificity for EV and proteins may result in sample contamination (Sidhom et al., 2020; Gurunathan et al., 2019).

Ultrafiltration (UF) is an emerging volume-exclusion-based technique that efficiently yields exosomes with high recovery rates and purities *via* membrane filters. However, lipid contamination during exosome precipitation remains difficult to avoid completely (Gurunathan et al., 2019; Coughlan et al., 2020). Size exclusion chromatography (SEC) offers significant advantages for exosome purification, effectively separating exosomes from most proteins and reducing contaminants or coprecipitates (de Freitas et al., 2021; Sidhom et al., 2020). SEC shows great promise for isolating pure exosomes from human body fluids, with potential for clinical and commercial applications. Nevertheless, SEC still faces challenges in accurately distinguishing exosomes from similarly sized vesicles and is limited by the volume of samples that can be processed per run. Emerging methods such as ExoTIC (Exosome Total Isolation Chip) (Liu et al., 2017), electric and acoustic field-based separation methods (Tayebi et al., 2021), and AC electrokinetic microarray chip technology (Ibsen et al., 2017) have shown great promise and are expected to further advance exosome research and applications.

Overall, while differential ultracentrifugation (UC) is still regarded as the “gold standard” for exosome isolation, factors such as sample purity, cost, efficiency, and operational complexity must be considered (Coughlan et al., 2020; Tian et al., 2020). The size and density similarities between exosomes and certain nonvesicular contaminants (e.g., lipoproteins and ribonucleoproteins) make their separation a major challenge (Huang et al., 2021; Singh et al., 2024). Therefore, future research should focus on developing simplified, cost-effective, and efficient isolation techniques that can preserve exosome activity and biological properties while effectively removing impurities (Witwer et al., 2021).

## 3 The role of mφ-exos in CVD

Mφ-Exos play crucial roles in the progression and treatment of CVD. They mediate intercellular communication and inflammation, influencing pathological processes such as and cardiac remodelling (Wu G. et al., 2020; Yang et al., 2022; Liu et al., 2020). Additionally, Mφ-Exos have potential as biomarkers for the early diagnosis of CVD and as targeted drug delivery systems to improve therapeutic outcomes (Han et al., 2022). Therefore, investigating the mechanisms of Mφ-Exos may offer new insights and strategies for diagnosing and treating CVD.

M1 and M2 macrophages play distinct roles in CVD (Mohd Idrus et al., 2021; Wang Z. et al., 2020). M1 macrophages are associated with proinflammatory responses, and their exosomes deliver various bioactive molecules that influence the function of surrounding cells, exacerbating inflammation and tissue damage (Liu et al., 2020). For example, proinflammatory factors carried by M1-Exos can accelerate AS, leading to endothelial dysfunction and cardiac remodelling (Barrett, 2020). In contrast, M2-type Mφ-Exos exhibit protective effects during cardiovascular disease progression. M2 macrophages are involved in anti-inflammatory responses and tissue repair, and their exosomes are rich in anti-inflammatory cytokines and growth factors, which promote vascular regeneration, alleviate inflammation, and improve cardiac function (Li L. et al., 2023). The miRNAs and proteins in M2-derived exosomes (M2-Exos) help regulate cardiac cell proliferation, survival, and fibrosis suppression, thereby slowing the progression of CVD (Wei and Zhao, 2022). Thus, studies on M2-Exos provide potential therapeutic strategies for CVD.

### 3.1 The Role of Mφ-Exos in Cardiovascular Inflammation

With increasing research, the potential of exosomes in modulating inflammation has gradually been revealed, highlighting their broad applications in CVD. Exosomes demonstrate dual potential in the precision diagnosis and treatment of cardiovascular diseases (CVD). In the diagnostic realm, their cargo of specific biomarkers—such as myocardial infarction-associated miRNAs (e.g., miR-1, miR-133a, miR-181) and heart failure-related proteins (e.g., NT-proBNP)—provides novel tools for early detection and prognostic evaluation (Crouser et al., 2021; Kuwabara et al., 2011; Moreira-Costa et al., 2021; Badacz et al., 2021; Lv B. et al., 2024). On the therapeutic front, stem cell-derived exosomes (e.g., mesenchymal stem cell exosomes) promote angiogenesis and myocardial regeneration by delivering reparative miRNAs like miR-21 and miR-210 (Shi et al., 2019; Wu Q. et al., 2020; Yang et al., 2024), while engineered exosomes further advance personalized therapies by targeting antifibrotic molecules (e.g., IMTP) (Wang et al., 2018) or inflammatory modulators (e.g., TGF-β inhibitors) (Ren et al., 2024).

**FIGURE 2:**
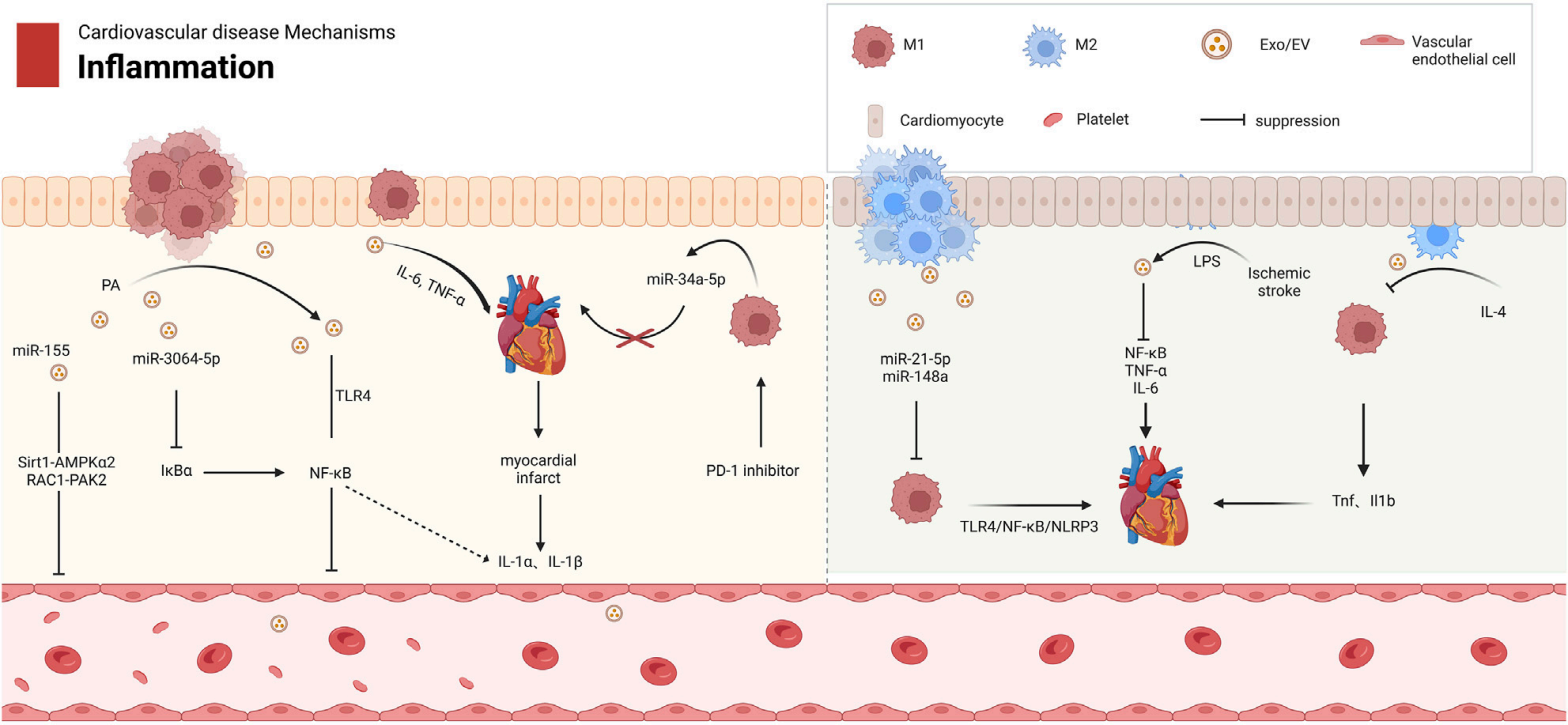
The Role of Mφ-Exos in Cardiovascular Inflammation. M1 and M2 macrophages have distinct roles in cardiovascular diseases. M1 exosomes release proinflammatory factors like miR-155 and TNF-α, activating pathways such as Sirt1-AMPKα2 and NF-κB, which inhibit endothelial cell growth and worsen cardiovascular disease. In contrast, M2 exosomes reduce inflammation by downregulating TNF-α and IL-1β in M1 macrophages. M2 exosomes also contain miR-21-5p and miR-148a, which regulate macrophage polarization and inhibit the TLR4/NF-κB/NLRP3 pathway, promoting tissue repair. AMPKα2 protein kinase AMP activated catalytic subunit alpha 2, EV extracellular vesicle, Exos Exosomes, IL Interleukin, LPS lipopolysaccharide, NF-κB nuclear factor κB, NLRP3 NACHT, LRR, PYD domain-containing protein 3, PAK2 p21 (RAC1)-activated kinase 2, PD-1 programmed cell death protein 1, RAC1 Rac family small GTPase 1, Sirt1 Sirtuin 1, TLR4 Toll-like receptor 4, TNF-α tumor necrosis factor-alpha.

Central to cardiovascular inflammation is the bidirectional regulation mediated by macrophage-derived exosomes. Pro-inflammatory M1 exosomes act as critical drivers of metabolic inflammation (metaflammation) by functioning as inflammatory mediators and metabolic regulators (Bhatnagar et al., 2007a; Huang-Doran et al., 2017; Chan et al., 2019; Bhatnagar et al., 2007b). These vesicles exacerbate injury through multiple pathways: releasing cytokines such as IL-6 and TNF-α to disrupt cardiovascular homeostasis (Sáez et al., 2019); impairing vascular repair via miR-155-dependent inhibition of fibroblast proliferation and activation of the RAC1-PAK2 and Sirt1-AMPKα2 signaling axis in endothelial cells (Liu et al., 2020; Y et al., 2024; S et al., 2020) (Figure 2); and amplifying myocardial inflammation through miR-34a-5p or NF-κB pathway activation under PD-1 inhibitor or palmitic acid stimulation (Xia et al., 2020; Dini et al., 2020; H et al., 2024) (Table 1). Furthermore, M1 exosomes enhance endothelial cytotoxicity by triggering the TLR4-NF-κB pathway (Liu et al., 2020; Biemmi et al., 2020).

**TABLE 1:** The role of macrophage extracellular vesicles in cardiovascular disease.

Exosomes	Mechanism	Role	Ref
Inflammatory response			
M1-derived miR-155	RAC1/PAK2 and Sirt1/AMPKα2	Impairs vascular repair	Liu et al. (2020), Y et al., 2024; S et al. (2020)
M1-derived miR-34a-5p	miR-34a-5p/PNUTS	Myocardial aging and inflammation	Xia et al. (2020), Dini et al. (2020), H et al. (2024)
M2-derived miR-21-5p	TLR4/NF-κB/NLRP3	Inhibits inflammation and promotes tissue repair	Li et al. (2023b), Tang et al. (2022), Dai et al. (2020)
M2-derived miR-148a			
M1-derived miR-155	M1-Exos depressed Sirt1/AMPK α2-endothelial nitric oxide synthase and RAC1/PAK2 signaling pathways	Reduces angiogenic capacity of ECs, exacerbates heart dysfunction in the myocardial infarction microenvironment. Inhibits angiogenesis and heart failure	Liu et al. (2020), He et al. (2024), Chen et al. (2021)
M1-derived miR-185-3p	Downregulates Smad7	Increases lipids, endothelial adhesion, oxidative stress, and inflammatory factors, promotes AS progression	Li et al. (2021)
M1-derived miR-222	miR-222/CDKN1B/CDKN1C pathway	Aggravates neointimal hyperplasia after carotid artery injury in mice, promotes acute coronary syndrome development	Wang et al. (2019)
M1-derived miR-663 b	Inhibits AMPK/Sirt1 signaling pathway	Induces proliferation, inflammation, oxidative stress, and migration, worsening PAH	Ma et al. (2023)
Angiogenesis			
M2-derived miR-132-3p	Downregulates THBS1	Promotes angiogenesis post-MI, promotes post-infarction angiogenesis	Guo et al. (2024a)
M2-derived miR-221-3p	Activates Grb10	Promotes HUVEC proliferation and inhibits apoptosis and inflammation, contributing to cardiovascular tissue repair	Cheng et al. (2022)
M1-derived miR-503	Inhibits IGF1R	Leads to endothelial cell dysfunction, severely impedes wound healing in diabetic patients	Wang et al. (2023)
Calcification and fibrosis			
M1-derived tsRNA-5006c	Mitochondrial autophagy	Regulates AVIC osteogenic differentiation, promotes calcific aortic valve disease	Xia et al. (2022)
M2-derived miR-17-5p	Inhibits TGF-β signaling pathway-induced VSMC osteogenic differentiation	Suppresses diabetic atherosclerotic plaque development	Baba et al. (2024)
M2-derived CircUbe3a	Targets miR-138-5p/RhoC axis	Promotes CF proliferation, migration, and phenotypic transformation, potentially exacerbating myocardial fibrosis post-acute myocardial infarction	Wang et al. (2021)
Cell death			
M2-derived miR-1271-5p	Downregulates SOX6	Reduces cardiomyocyte apoptosis, promotes cardiac repair	Long et al. (2021)
M2-derived Exos	Activates Nrf2/HO-1 signaling pathway	Reduces oxidative damage-induced cell death after ischemic stroke	Xiao et al. (2022)
M1-derived miR-16-5p	Inhibits SMAD7	Induces apoptosis and exacerbates AS progression	Chen et al. (2022)
THP-1 macrophage-derived lncRNA LIPCAR	Unexplored	Inhibits HUVEC and vascular smooth muscle cell apoptosis, alleviates cardiovascular cell damage	Hu et al. (2021)
M1-derived lncRNA PVT1	Inhibits miR-186-5p and HMGB1	Promotes inflammation and pyroptosis in abdominal aortic aneurysm vascular smooth muscle cells	Zhang et al. (2024a)
M2-derived Exos	Inhibits oxidative stress and NLRP3 pathway	Reduces cardiomyocyte pyroptosis, improves I/R injury	Hu et al. (2022)
M2-derived miR-148a	Inhibits TXNIP/TLR4/NF-κB/NLRP3 pyroptosis signaling pathway	Alleviates MI/R injury	Dai et al. (2020)
M2-derived miR-378a-3p	Blocks NLRP3/Caspase-1/GSDMD pathway	Reduces cardiomyocyte pyroptosis, improves cardiac function	Dai et al. (2020), Yuan et al. (2022)
M2-derived lncRNA AK083884	Inhibits PKM2 binding with HIF-1α	Promotes macrophage metabolic reprogramming and M2 polarization, ultimately improving symptoms of viral myocarditis	Zhang et al. (2024b)
M2-derived miR-181b-5p	Reduces glucose uptake and glycolysis	Reduces mtROS production, improves cardiac function	Li et al. (2023a)
M2-derived GDF15	Activates p-Smad2/FABP4 signaling pathway	Reprograms macrophages and enhances myocardial repair	Xiao et al. (2024)

In contrast, anti-inflammatory M2 exosomes suppress inflammation and promote tissue repair through two distinct mechanisms: polarizing macrophages toward a reparative phenotype via miR-21-5p or blocking the TLR4/NF-κB/NLRP3 inflammasome cascade via miR-148a (Li Q. et al., 2023; Tang et al., 2022; Dai et al., 2020) (Table 1). Importantly, external interventions such as IL-4 induction or LPS preconditioning can amplify the anti-inflammatory efficacy of M2 exosomes, offering targeted therapeutic opportunities for ischemic heart disease (Phu et al., 2022; Zheng et al., 2019).

Despite these advances, significant challenges remain. The identification of macrophage exosomes-specific inflammatory biomarkers and their corresponding therapeutic strategies remains limited. To bridge this gap, future studies must integrate single-cell sequencing and spatial multi-omics technologies to dissect the interplay between exosomes and the immune microenvironment, thereby accelerating the development of targeted interventions for CVD. The identification of macrophage exosome-specific inflammatory biomarkers and their corresponding therapeutic strategies remains limited. For instance, current assays exhibit low sensitivity in detecting critical miRNAs such as miR-208a and miR-499 in acute myocardial infarction (MI) patients (Crouser et al., 2021), and cTnT fails to diagnose unstable angina due to its reliance on irreversible myocardial necrosis (Kuwabara et al., 2011). Additionally, the mechanisms underlying M2 exosome-mediated cardioprotection (e.g., AK083884/SOCS2/PKM2 signaling in viral myocarditis) remain unclear (Liu et al., 2020; Y et al., 2024). Moreover, many experimental models do not fully replicate the complexities of human clinical conditions. In future studies, we aim to develop more physiologically relevant models (e.g., hypoxia models or in-depth clinical trials) and focus on improving exosome isolation and detection sensitivity while elucidating the unclear molecular mechanisms of M2 exosomes. Regardless, the current findings provide new insights into the clinical application of exosomes for treating CVD. We hope to conduct further research to validate these findings and develop additional therapeutic strategies.

### 3.2 The Role of Mφ-exos in angiogenesis regulation in CVD

**FIGURE 3:**
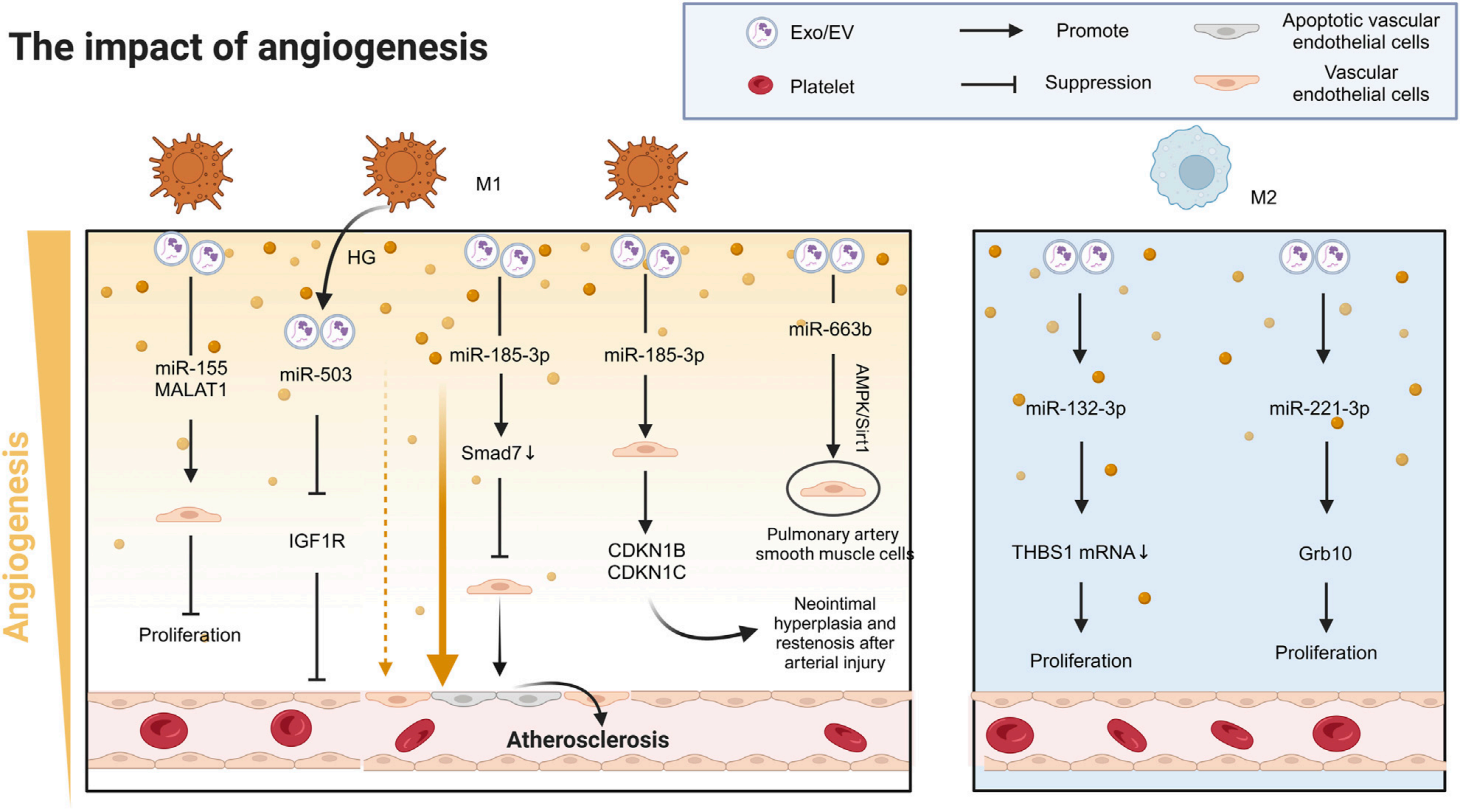
The role of Mφ-Exos in cardiovascular angiogenesis. In MI, M1 exosomes release miR-155 and MALAT1, reducing endothelial cell angiogenesis and worsening heart dysfunction. M1 exosomes also contribute to cardiovascular disease *via* miR-503 and miR-185-3p, which inhibit endothelial cell growth and promote atherosclerosis. miR-222 from M1 exosomes causes arterial restenosis. In contrast, M2 exosomes release miR-132-3p and miR-221-3p, enhancing endothelial cell growth and angiogenesis, improving heart function. AMPK/Sirt1 Adenosine 5‘-monophosphate (AMP)-activated protein kinase/Sirtuin 1, CDKN1B/C cyclin-dependent kinase inhibitor 1B/C, EV Extracellular vesicle, Exos exsomes, Grb10 Growth factor receptor binding protein-10, HG High glucose, IGF1R Insulin-like growth factor 1 receptors, MALAT1 Metastasis Associated Lung Adenocarcinoma Transcript 1, MI Myocardial infarction, Smad7 Small mother against decapentaplegic-7, THBS1 Thrombospondin-1.

In the context of myocardial infarction (MI), the release of miR-155 and MALAT1 from M1-Exos significantly reduces the angiogenic capacity of endothelial cells (ECs), exacerbating heart dysfunction in the microenvironment of myocardial infarction (Liu et al., 2020; He et al., 2024; Chen et al., 2021). Beyond this mechanism, M1-Exos drive CVD progression through pleiotropic signaling pathways that disrupt cellular homeostasis across diverse cardiovascular cell types. For example, miR-185-3p suppresses endothelial cell proliferation and induces apoptosis by downregulating small mothers against decapentaplegic-7 (Smad7), promoting the pathological progression of AS (Li et al., 2021). Similarly, miR-222 regulates the expression of cyclin-dependent kinase inhibitor 1 B (CDKN1B) and CDKN1C in vascular smooth muscle cells (VSMCs), facilitating neointimal hyperplasia and restenosis after arterial injury (Wang et al., 2019) (Figure 3). In pulmonary arterial hypertension (PAH), miR-663 b in M1-Exos inhibits the AMPK/Sirt1 signalling pathway in pulmonary artery smooth muscle cells (PASMCs), inducing proliferation, inflammation, oxidative stress, and migration, thereby worsening the disease condition (Ma et al., 2023) (Table 1).

In stark contrast to the proinflammatory effects of M1-Exos, M2-Exos display remarkable proangiogenic effects in cardiovascular repair. Following MI, M2-Exos transfer miR-132-3p to endothelial cells, downregulating thrombospondin-1 (THBS1) mRNA expression, significantly promoting angiogenesis and improving cardiac function (Guo H. et al., 2024). Moreover, miR-221-3p in M2-Exos targets growth factor receptor binding protein-10 (Grb10), promoting HUVEC proliferation and inhibiting apoptosis and inflammation, thereby contributing to cardiovascular tissue repair (Cheng et al., 2022) (Figure 3) (Table 1). Furthermore, large EV derived from human regulatory macrophages (L-EVMregs) have shown potential in regulating wound healing and angiogenesis (Albrecht et al., 2023a; Albrecht et al., 2023b).

Notably, diabetic foot ulcers (DFUs), significant complications of CVD, are closely associated with metabolic disturbances, inflammatory responses, and vascular damage induced by hyperglycemia (Chin et al., 2024). The activation of M1 macrophages and the release of proinflammatory factors play critical roles in the development and progression of DFUs (Lin et al., 2022). Studies have demonstrated that under high-glucose (HG) conditions, M1-Exos inhibit IGF1R in HUVECs via miR-503, leading to endothelial cell dysfunction and significantly impeding wound healing in diabetic patients (Wang et al., 2023) (Figure 3) (Table 1).

In conclusion, macrophage-derived exosomes exhibit a dynamic “disruption-repair” equilibrium in cardiovascular angiogenesis through the bidirectional regulation of M1 and M2 subtypes. Targeting these exosomes holds promise for precision therapies in myocardial infarction, pulmonary arterial hypertension, and diabetes-related vascular complications. However, advancing these therapies requires deeper exploration of exosomal heterogeneity and the development of spatiotemporally specific modulation strategies, such as single-exosome analysis (Guo et al., 2023), spatial multi-omics (Vandereyken et al., 2023), and microfluidic chip-based technologies (Mun et al., 2023).

### 3.3 Dual regulatory role of Mφ-Exos in Vascular Calcification and Myocardial Fibrosis

Mφ-Exos play pivotal roles in two critical pathological processes of CVD—vascular calcification and myocardial fibrosis—by regulating inflammatory factors and signalling pathways.

#### 3.3.1 The Role of Mφ-exos in vascular calcification

**FIGURE 4:**
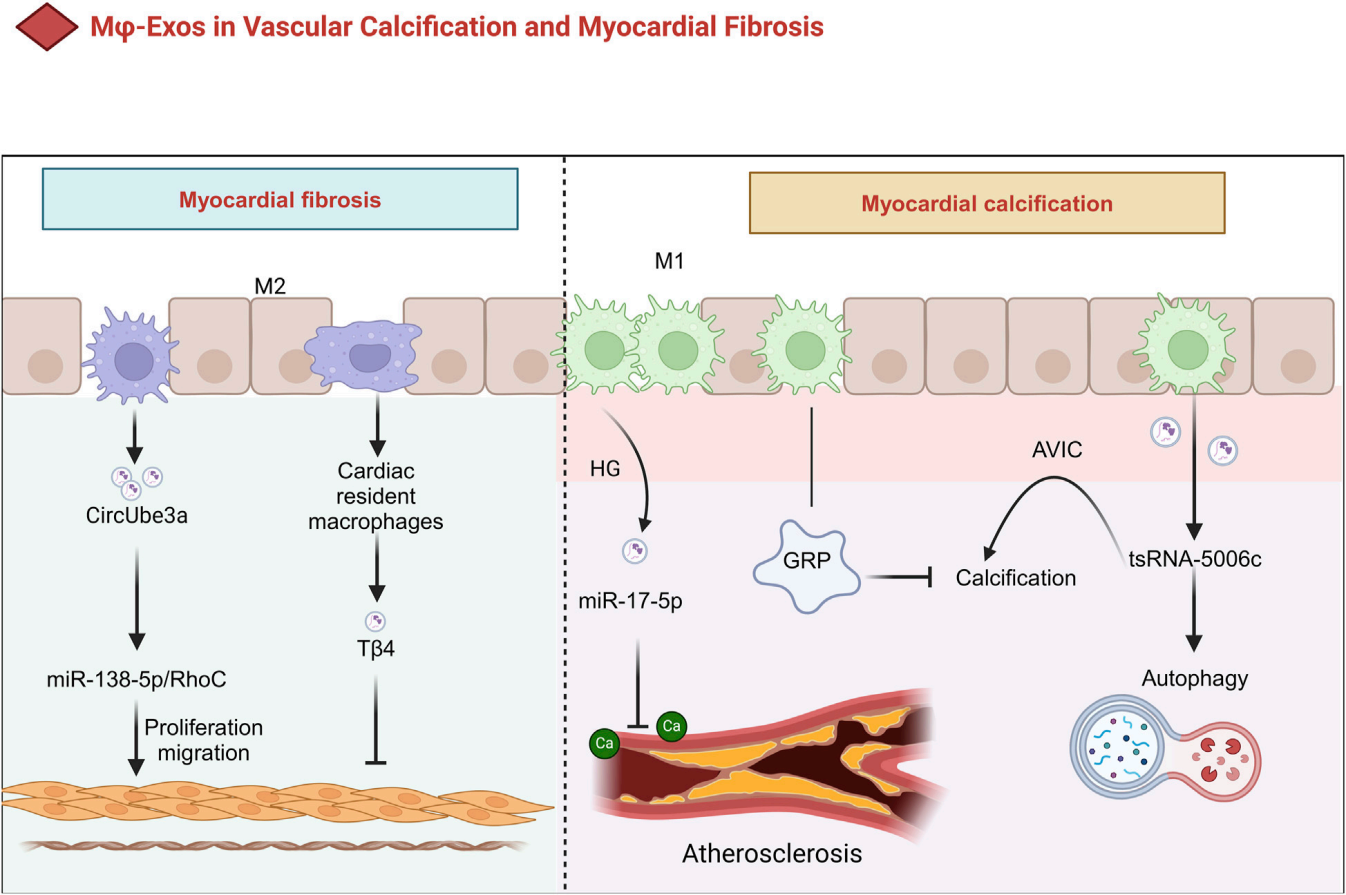
The Role of Mφ-Exos in Vascular Calcification and Myocardial Fibrosis. In HG conditions, Mφ exosomes contain miR-17-5p, which inhibits calcification in diabetic atherosclerotic plaques. They also carry GRP, reducing calcification in cardiovascular and joint tissues via anti-inflammatory effects. M1 exosomes promote calcific aortic valve disease through tsRNA-5006c-regulated autophagy. Meanwhile, M2-sEVs drive cardiac fibroblast proliferation and migration via CircUbe3a, contributing to myocardial fibrosis after acute myocardial infarction. AVIC Aortic valve interstitial cell, GRP Gla-rich protein; HG high glucose, Tβ4 Thymosin β4, sEVs small extracellular vesicles.

Vascular calcification is an independent risk factor for cardiovascular disease progression, significantly increasing the risk of all-cause mortality and atherosclerotic plaque rupture through multiple biological mechanisms. Its primary inducers include aging, elevated serum calcium and phosphate levels, inflammation, and oxidative stress, which may ultimately lead to hypertension and heart failure (Yaker et al., 2022; Kurabayashi, 2019). During apoptosis, matrix vesicles released by macrophages and vascular smooth muscle cells (VSMCs) play a key role in the formation of fine-particle calcification. For example, macrophage-derived matrix vesicles containing high-calcification-potential molecules such as CD9, TSG101, and S100A9 contribute to microcalcification in the carotid artery intima (New et al., 2013; McCormick et al., 2005). Moreover, macrophage apoptosis is associated with large, punctate calcifications, whereas VSMC apoptosis results in small microcalcifications (New et al., 2013) (Figure 4).

M1-Exos regulate the calcification process by delivering noncoding RNAs (e.g., miRNAs or tsRNAs). For example, M1-EV modulate the osteogenic differentiation of aortic valve interstitial cells (AVICs) through autophagy pathways driven by tsRNA-5006c, a process critical to the progression of calcific aortic valve disease (Xia et al., 2022). Additionally, under high-glucose (HG) conditions, miR-17-5p levels increase significantly in Mφ-EV, which inhibits calcification within atherosclerotic plaques in diabetic patients (Baba et al., 2024) (Table 1). On the other hand, Gla-rich protein (GRP), a calcification inhibitor, may reduce calcification in cardiovascular and joint tissues through anti-inflammatory mechanisms. Interestingly, GRP has been detected at both the protein and mRNA levels in macrophage exosomes, suggesting that Mφ-Exos may serve as carriers for their extracellular transport and release (Viegas et al., 2017; Dhore et al., 2001) (Figure 4).

#### 3.3.2 The Role of Mφ-exos in myocardial fibrosis

Small EV from M2 macrophages mediate myocardial fibrosis following acute MI through CircUbe3a, which directly targets the miR-138-5p/RhoC axis to promote the proliferation, migration, and phenotypic transformation of cardiac fibroblasts (CFs) (Table 1) (Wang et al., 2021). Furthermore, exosomes derived from cardiac-resident macrophages modified with thymosin β4 (Tβ4) have been shown to significantly reduce myocardial fibrosis, providing a novel strategy for cardiovascular disease treatment (Chen et al., 2023) (Figure 4).

Elucidating the properties of Mφ-Exos under normal and pathological conditions is not only essential for the prevention and treatment of vascular calcification but may also offer new therapeutic approaches for CVD (Zazzeroni et al., 2018). By further investigating these mechanisms, innovative treatment strategies can be developed, ultimately improving CVD clinical outcomes (Figure 4).

While elucidating the properties of macrophage-derived exosomes (Mφ-Exos) under normal and pathological conditions holds promise for advancing therapies targeting vascular calcification and CVD (Zazzeroni et al., 2018), critical challenges and unresolved questions remain. First, the dual roles of Mφ-Exos in myocardial fibrosis highlight the context-dependent heterogeneity of exosomal functions, which complicates therapeutic targeting. Second, current research primarily relies on preclinical models, leaving significant gaps in understanding exosomal behavior in human pathophysiology, such as patient-specific variations in immune microenvironment interactions. Finally, strategies to achieve spatiotemporal control of Mφ-Exos delivery or mitigate off-target effects (e.g., the inability of exosomes to precisely release drugs in injured heart regions. Or the unintended activation of normal fibroblasts, which exacerbates fibrosis remain underexplored. Addressing these limitations through mechanistic studies and standardized protocols is essential for leveraging Mφ-Exos to improve CVD clinical outcomes (Figure 4).

### 3.4 Role of mφ-exos in lipid metabolism and CVD

**FIGURE 5:**
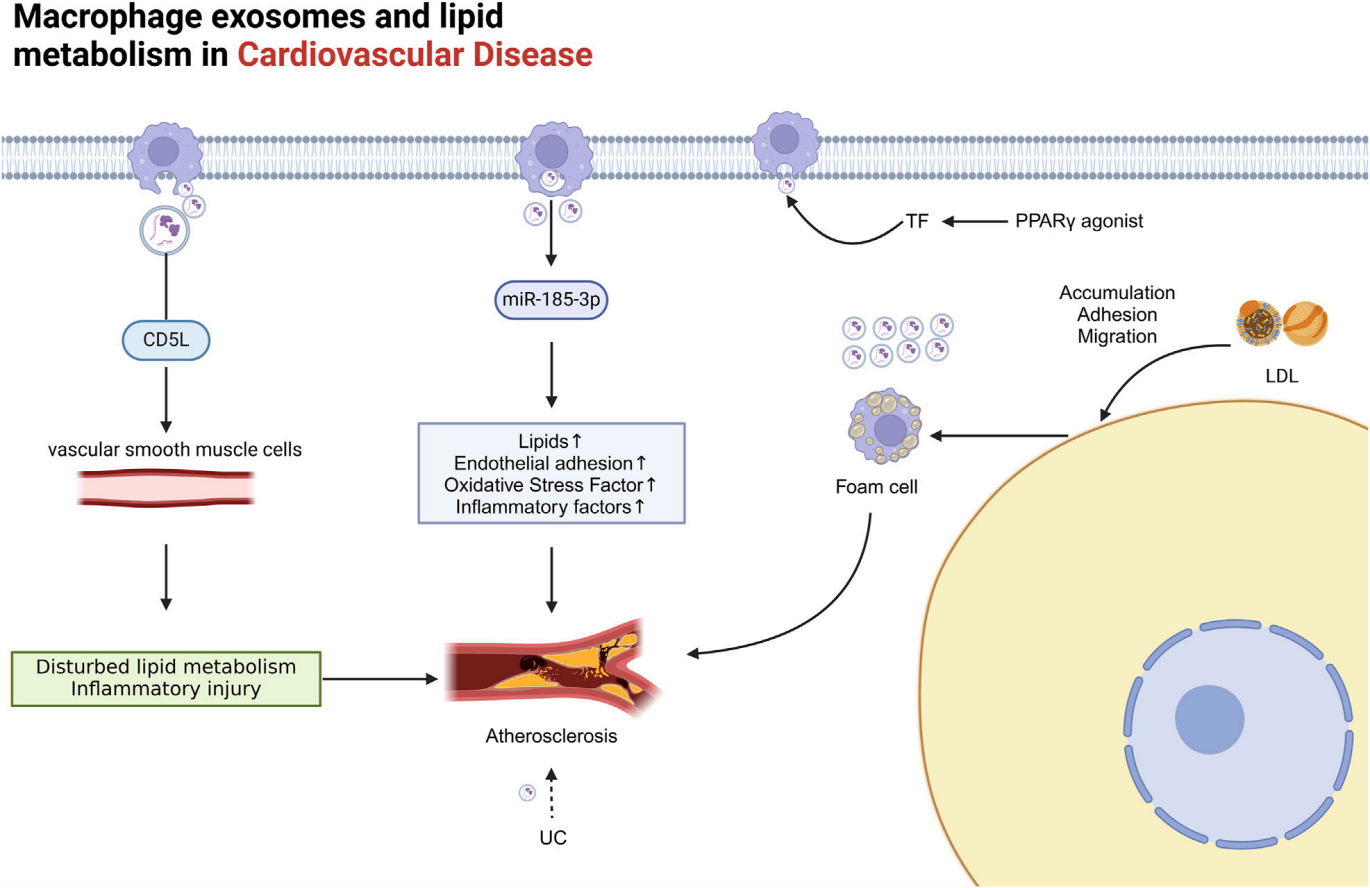
The role of Mφ-Exos in cardiovascular lipid metabolism Mφ exosomes significantly impact metabolic disorders. M1 exosomes contain miR-185-3p, which raises blood lipid levels and endothelial adhesion, while CD5L protein in these exosomes promotes atherosclerosis by regulating lipid metabolism in vascular smooth muscle cells. Accumulation of UC or rosiglitazone boosts vesicle and Mφ exosome secretion. LDL buildup increases macrophage adhesion to damaged endothelial cells, forming foam cells, which release more exosomes than normal macrophages, further worsening cardiovascular disease. AS atherosclerosis, CD5L CD5-like molecule, Exosome Exos, LDL low-density lipoprotein, PPARγ, TF tissue factor, UC unesterified cholesterol.

Exosomes have significant impacts on metabolic disorders (Castaño et al., 2018). They are enriched with specific lipids, such as sphingolipids, cholesterol, and phosphatidylserine, which exhibit high lipid order and greater resistance to detergents (Record et al., 2018). These lipids not only play crucial roles in the biological functions of exosomes but also alter the phenotypes of recipient cells (Skotland et al., 2017). Additionally, exosomes serve as potent sources of eicosanoids, such as prostaglandins and leukotrienes, which exhibit bioactivity both *in vivo* and *in vitro* (Boilard, 2018) and regulate the expression of classical lipid transport proteins, such as ABCA1-mediated cholesterol efflux (Stamatikos et al., 2020). Exosomes also participate in lipid degradation and redistribution within adipose tissue, reducing lipid accumulation and improving cardiac function (Zhou et al., 2020) (Figure 5).

Mφ-Exos play an essential role in CVD, particularly AS, by regulating lipid metabolism and signalling pathways. During the pathological process of AS, exosome-mediated lipid metabolism involves the synthesis, transport, and degradation of lipids (Castaño et al., 2018; Wang W. et al., 2020). Studies have shown that elevated levels of miR-185-3p in M1-derived exosomes promote AS progression, increasing blood lipids, endothelial adhesion, oxidative stress, and inflammatory factors (Li et al., 2021). Additionally, the CD5-like molecule (CD5L) protein in macrophage exosomes can induce inflammation and vascular smooth muscle cell lipid dysregulation, promoting AS progression (Wang L. et al., 2022). The accumulation of unesterified cholesterol (UC) further triggers the release of procoagulant microvesicles by monocytes and macrophages, exacerbating the lesion (Liu et al., 2012). The use of PPARγ agonists, such as rosiglitazone, and certain prostaglandins can increase the release of tissue factor (TF)-rich microvesicles from monocytes and macrophages (Neri et al., 2012) (Figure 5).

AS formation involves the accumulation of low-density lipoprotein (LDL) particles in the intima, adhesion, migration, and maturation of monocytes on damaged endothelial cells, ultimately leading to the formation of foam cells (Libby et al., 2011). Macrophage-derived foam cells, which are key contributors to AS development, release significantly more exosomes than normal macrophages do (Gutierrez, 2022; Niu et al., 2016). These exosomes not only promote AS progression but also impact key steps in lipid metabolism (Figure 5).

Deciphering the spatiotemporal dynamics of Mφ-Exos in lipid metabolism—from foam cell communication to systemic lipid redistribution—will not only refine our understanding of CVD progression but also pave the way for exosome-guided precision therapies, such as engineered vesicles for targeted drug delivery or liquid biopsies for early AS detection.

### 3.5 Mφ-exos regulate cardiovascular cell death mechanisms

Mφ-Exos play critical roles in the development and progression of CVD by regulating various cell death mechanisms, including apoptosis, pyroptosis, and ferroptosis. These processes highlight the essential role of Mφ-Exos in pathological events and provide potential therapeutic strategies for associated diseases.

#### 3.5.1 The role of mφ-exos in regulating apoptosis

Apoptosis is a common form of cell death in cardiovascular disease, where Mφ-Exos can exhibit both protective and detrimental effects.

Studies have shown that M2-Exos carrying miR-1271-5p reduce cardiomyocyte apoptosis by downregulating SOX6, thereby promoting cardiac repair (Long et al., 2021). Additionally, M2-Exos activate the Nrf2/HO-1 signalling pathway in oxygen–glucose deprivation/reoxygenation (OGD/R)-treated HT22 neuronal cells, reducing oxidative damage after ischemic stroke (Xiao et al., 2022). In contrast, miR-16-5p in M1-derived exosomes aggravates AS progression and induces apoptosis by downregulating SMAD7 expression (Chen et al., 2022). Interestingly, the lncRNA LIPCAR in exosomes released by ox-LDL-treated THP-1 macrophages (THP-1/ox-LDL Exos) inhibits the apoptosis of HUVECs and vascular smooth muscle cells, thereby mitigating cardiovascular cell damage (Hu et al., 2021) (Table 1).

Mφ-Exos have also shown neuroprotective potential. For example, exosomes secreted by LPS-stimulated RAW264.7 macrophages (LPS-Exs) enhance the M2 polarization of microglia, reduce neuronal apoptosis, decrease infarct size, and improve neurological function, providing new insights for ischemic stroke treatment (Zheng et al., 2019).

#### 3.5.2 The Role of Mφ-exos in pyroptosis regulation

Pyroptosis, an inflammatory form of cell death, plays a significant role in CVD. Mφ-Exos regulate pyroptosis through multiple molecular mechanisms, influencing disease progression.

The lncRNA PVT1 in M1-derived exosomes promotes inflammation and pyroptosis of vascular smooth muscle cells in abdominal aortic aneurysms by inhibiting miR-186-5p and regulating HMGB1 (Zhang J. et al., 2024) (Table 1). Furthermore, tissue factors released during macrophage pyroptosis enter the bloodstream as microvesicles, triggering coagulation and causing organ damage (Wu et al., 2019). Pyroptotic cell-derived microparticles also play critical roles in cardiovascular disease progression (Sun et al., 2021).

Conversely, M2-Exos exert protective effects by inhibiting pyroptosis. M2-Exos significantly reduce cardiomyocyte pyroptosis, particularly in ischemia‒reperfusion (I/R) injury, by suppressing oxidative stress and the NLRP3 pathway (Hu et al., 2022). miR-148a in M2-Exos alleviates myocardial ischemia/reperfusion (MI/R) injury by inhibiting the TXNIP-TLR4/NF-κB/NLRP3 pyroptosis signalling pathway (Dai et al., 2020). Additionally, miR-378a-3p in M2-EV blocks the NLRP3/Caspase-1/GSDMD pathway, further reducing cardiomyocyte pyroptosis (Dai et al., 2020; Yuan et al., 2022) (Table 1).

During AS pathology, Mφ-Exos suppress pyroptosis to exert protective effects. For example, EV prevent foam cell formation and macrophage pyroptosis by inhibiting HMGB1 release and Caspase-11 activation, thereby slowing plaque formation in mice (Liang et al., 2024). Exosomes carrying miR-199a-5p inhibit the SMARCA4/PODXL/NF-κB axis, reducing endothelial cell pyroptosis and preventing AS progression (Liang et al., 2023).

#### 3.5.3 The Role of Mφ-exos in ferroptosis regulation

Ferroptosis, a novel cell death mechanism, is closely associated with CVD, such as MI. Iron overload and ferroptosis significantly contribute to pathological deterioration and poor prognosis in MI patients (Miao et al., 2023).

Studies have demonstrated that Mφ-Exos act as endogenous bioiron chelators by regulating iron homeostasis and delivering antioxidants, such as glutathione, effectively reducing iron overload and suppressing ferroptosis in cardiomyocytes (Guo D. et al., 2024). Iron chelators such as deferoxamine and iron carrier proteins have also shown therapeutic potential in ferroptosis-related treatments, possibly working in concert with Mφ-Exos to improve cardiac dysfunction following MI (Wang and Kang, 2021; Wang Z. et al., 2022).

Mφ-Exos play dual roles in cardiovascular cell death regulation, with M2-Exos often exerting protective effects (e.g., miR-1271-5p reducing apoptosis (Long et al., 2021), miR-148a suppressing pyroptosis (Dai et al., 2020) and M1-Exos driving pathology (e.g., miR-16-5p inducing apoptosis (Chen et al., 2022), PVT1 promoting pyroptosis (Zhang J. et al., 2024). Their ability to modulate ferroptosis through iron chelation and antioxidant delivery further underscores their therapeutic potential (Guo D. et al., 2024; Wang and Kang, 2021; Wang Z. et al., 2022). However, challenges such as EV specificity (For instance, CRISPR-based editing could optimize exosomal miRNA profiles to minimize off-target effects), pathological context dependency, and translational barriers (Current models often fail to replicate human-specific exosome heterogeneity) must be addressed. Future efforts should focus on engineering exosomes for precision delivery, integrating multi-omics to map human-specific mechanisms, and developing Exo related combination therapies to optimize clinical outcomes.

### 3.6 Regulation of cardiovascular cell reprogramming by mφ-exos

M2-Exos play a pivotal role in reprogramming target cells during CVD, facilitating cardiac repair and regeneration. The underlying mechanisms involve primarily inflammation modulation, cell migration, and regeneration. The activation and polarization of proinflammatory macrophages are closely associated with their metabolic patterns, which rely heavily on glycolysis for energy production (O'Neill and Hardie, 2013; Colegio et al., 2014). Studies have shown that both tissue-resident and monocyte-derived proinflammatory macrophages exhibit significantly elevated glycolytic activity (Nathan and Cunningham-Bussel, 2013; Chen Z. et al., 2024). High glucose uptake promotes a metabolic shift toward lactate production, accelerating ATP synthesis while increasing the level of mitochondrial reactive oxygen species (mtROS) (Shirai et al., 2016). An increase in mtROS further increases the expression and release of inflammatory factors (Nathan and Cunningham-Bussel, 2013). This metabolic change is particularly pronounced in coronary artery disease (CAD) patients, highlighting the need for strict monitoring of glucose intake (Nathan and Cunningham-Bussel, 2013).

Compared with proinflammatory macrophages, M2-Exos promote anti-inflammatory polarization and metabolic reprogramming of macrophages, thus facilitating tissue repair. For example, M2-Exos reduce ROS accumulation to alleviate oxidative stress and mitigate hypoxia/reoxygenation (H/R)-induced cell injury (Hu et al., 2022). Additionally, the lncRNA AK083884 in M2-Exos interacts with macrophage pyruvate kinase M2 (PKM2), inhibiting its binding to HIF-1α and thereby promoting macrophage metabolic reprogramming and M2 polarization, ultimately improving the symptoms of viral myocarditis (Zhang Y. et al., 2024) (Table 1). M2-EV also exhibit strong immunomodulatory potential, regulating the cardiac immune microenvironment for enhanced heart repair; however, their limited *in vivo* targeting capability poses therapeutic challenges (Pei et al., 2024).

M2-EV encapsulate specific microRNAs (e.g., miR-181b-5p, miR-21/99a/146b/378a, and miR-33) that mediate macrophage metabolism (Li L. et al., 2023; Phu et al., 2022). For example, miR-181b-5p in M2-EV reduces macrophage glucose uptake and glycolysis after acute MI, decreasing mtROS production to improve cardiac function and repair while regulating CCR2 macrophage activity (Li L. et al., 2023). Furthermore, macrophage-derived exosomes can deliver growth differentiation factor 15 (GDF15), activating the p-Smad2/3-FABP4 signalling pathway to reprogram macrophages and enhance myocardial repair (Xiao et al., 2024) (Table 1). Recent studies have indicated that exosomes derived from IL-4-stimulated THP-1 macrophages (THP-1-IL4-Exos) upregulate miR-21/99a/146b/378a and downregulate miR-33 expression, increasing fatty acid uptake, mitochondrial activity, and oxidative phosphorylation (OXPHOS). These exosomes reprogram macrophage metabolism, polarize them to an anti-inflammatory phenotype, and mitigate inflammation in the aorta, adipose tissue, and liver (Phu et al., 2022).

Metabolic reprogramming can reverse energy imbalances in pathological states, regulate macrophage polarization phenotypes, and shift the cellular response from inflammation-driven damage to tissue repair, offering novel therapeutic strategies for cardiovascular diseases (CVD). These effects may be driven by Mφ-Exos through mechanisms such as miR-181b-5p-mediated reduction of glycolysis (Li L. et al., 2023), AK083884-driven PKM2/HIF-1α interactions (Zhang Y. et al., 2024), and GDF15-activated Smad2/3-FABP4 signaling (Xiao et al., 2024). However, challenges such as limited *in vivo* targeting efficiency (Pei et al., 2024) and patient-specific metabolic heterogeneity (Nathan and Cunningham-Bussel, 2013) must be addressed to fully realize their clinical potential.

## 4 Insights into the therapeutic potential of mφ-exos

Exosomes, as efficient delivery vehicles, can carry small molecule drugs, biological agents (e.g., siRNAs, miRNAs), and cytokines, demonstrating immense potential for disease therapy. The targeted delivery of therapeutic agents *via* exosomes can improve cardiac function, reduce myocardial injury, and decrease the incidence of cardiac fibrosis (Kang et al., 2023).

In CVD, molecularly engineered M2-Exos exhibit potent anti-inflammatory properties and therapeutic potential against AS. For example, molecularly engineered M2-Exos with an inflammatory response and intrinsic heme biosynthesis capabilities enable both imaging and treatment of AS, significantly alleviating disease progression (Wu G. et al., 2020). Additionally, M2-Exos fused with platelet membranes (P-M2 EV) target atherosclerotic plaques, inhibiting foam cell formation and endothelial cell inflammation while reducing plaque formation and progression. This effect is achieved through the delivery of anti-inflammatory miRNAs (e.g., miR-99a-5p) and anti-inflammatory proteins (Xie et al., 2024). In viral myocarditis, M2-EV engineered with cardiac-targeting peptides (CTPs) and platelet membranes (PMs) enhance their targeting ability, promote M1-to-M2 macrophage polarization, protectively regulate the cardiac immune microenvironment, and facilitate cardiac repair (Pei et al., 2024).

Exosomes also show potential in treating ischemic brain injury and supporting neuronal recovery. Functionalized M2 nanomedicines downregulate NF-κB signalling pathways and inflammatory cytokines (TNF-α and IL-6) in microglia poststroke, promoting neuronal recovery (Lv Z. et al., 2024). Furthermore, curcumin (Cur) encapsulated in Mφ-Exos targets ischemic regions, inhibits ROS-mediated mitochondrial apoptosis and alleviates ischemia/reperfusion (I/R) injury (He et al., 2020).

Although M1-derived exosomes (M1-Exos) typically exacerbate cardiovascular disease pathology, they can also be harnessed for therapeutic purposes through rational design. For example, M1-Exos loaded with edaravone (Edv) significantly enhance drug bioavailability and half-life, improving drug targeting to ischemic regions. This approach reduces cell death and promotes the beneficial polarization of inflammatory cells. Recent studies have demonstrated that Edv-loaded exosomes (Exo + Edv) reduce neuronal death and facilitate microglial polarization from the M1 phenotype to the M2 phenotype poststroke, revealing their potential in treating both neurological diseases and CVD (He et al., 2020).

In conclusion, exosomes serve as novel drug delivery platforms that exhibit multifunctional therapeutic capabilities in cardiovascular and neurological diseases, offering innovative research directions for precision treatment of complex conditions.

## 5 Conclusions and future perspectives

Mφ-Exos, as critical mediators of intercellular communication, have garnered increasing attention for their multifunctionality and potential clinical applications in complex pathologies such as CVD. In recent years, significant progress has been made in understanding their roles in disease diagnosis and treatment, particularly in immune regulation, metabolic reprogramming, and therapeutic molecule delivery.

In diagnostics, exosomes, as carriers of proteins and nucleic acids (e.g., miRNAs, long noncoding RNAs (lncRNAs), circular RNAs (circRNAs)) derived from specific cell types, offer novel biomarker opportunities. These cargo molecules not only reflect disease states but also serve as potential therapeutic targets. For example, changes in the expression levels of AS-associated miRNAs, such as miR-155, miR-21, and miR-126, demonstrate their diagnostic potential. Similarly, altered exosome molecular profiles in diabetes and coronary artery disease patients highlight their sensitivity in monitoring disease progression. These properties (Such as miR-503 miR-17-5p、TsRNA-5006c, They are closely related to endothelial cell disorder, diabetes related atherosclerosis, and vascular calcification.) not only enable early disease detection but also provide critical insights for designing personalized treatment strategies.

In therapeutics, engineered exosomes have achieved precise delivery to diseased tissues. For example, exosomes encapsulating anti-inflammatory miRNAs or curcumin can effectively alleviate inflammation and apoptosis. Membrane-modification strategies, such as platelet membrane fusion or cardiac-targeting peptide functionalization, further increase the targeting efficiency of exosomes, offering promising therapeutic solutions for CVD and stroke. However, challenges remain in large-scale exosome production and quality control, particularly regarding functional consistency, safety, and long-term stability. Optimizing the manufacturing and purification processes will be essential to ensure their clinical feasibility.

Furthermore, the distribution and functional diversity of macrophage subtypes play pivotal roles in CVD progression. For example, the M2a, M2b, and M2c subtypes play distinct roles in tissue repair, immune regulation, and inflammation control. Foam cells and CCR2 macrophages secrete exosomes that regulate the pathological processes of AS and myocarditis, respectively. This highlights the unique value of exosomes derived from different macrophage subtypes in modulating specific disease pathways. Additionally, investigating the secretion mechanisms of exosomes and their functional dynamics under various pathological conditions will further clarify their roles in disease progression.

Future research on macrophage-derived exosomes (Mφ-Exos) in cardiovascular diseases (CVD) should focus on elucidating their roles in cell subtype-specific regulation, inflammation modulation, proliferation, cell death, and metabolic reprogramming. First, the functional heterogeneity of macrophage subtypes (e.g., M2a, M2b, M2c) and their exosomal cargo must be systematically mapped to understand their distinct contributions to tissue repair, immune regulation, and inflammatory control. For instance, how do foam cell-derived exosomes differ from CCR2 macrophage exosomes in regulating atherosclerosis (AS) and myocarditis? Second, the interplay between Mφ-Exos and cell death pathways (e.g., apoptosis, pyroptosis, ferroptosis) requires deeper exploration. For example, can M2-Exos be engineered to selectively inhibit NLRP3-mediated pyroptosis while preserving beneficial apoptosis in ischemic injury? Third, the role of Mφ-Exos in metabolic reprogramming—such as miR-181b-5p-mediated glycolysis suppression (Li L. et al., 2023) or AK083884-driven fatty acid oxidation (Zhang Y. et al., 2024)—should be further dissected to optimize their therapeutic potential.

Technological advancements will be critical to these efforts. Integrating single-cell multi-omics (e.g., transcriptomics, proteomics) with spatial imaging can reveal exosome-mediated crosstalk in the cardiovascular microenvironment. Engineering exosomes with stimuli-responsive properties (e.g., pH- or ROS-sensitive release) and tissue-specific targeting (e.g., cardiac homing peptides) will enhance their precision and efficacy. Additionally, combining Mφ-Exos with existing therapies (e.g., iron chelators for ferroptosis, NLRP3 inhibitors for pyroptosis) may unlock synergistic benefits for complex CVD pathologies.
